# Health Literacy Among Patients Evaluated for Drug Hypersensitivity in a Tertiary Allergy Clinic: A Cross-Sectional Study

**DOI:** 10.3390/medicina62102024

**Published:** 2026-10-19

**Authors:** Begum Gorgulu Akin, Makbule Seda Bayrak Durmaz, Fikriye Kalkan, Sadan Soyyigit

**Affiliations:** 1 Department of Allergy and Clinical Immunology, Faculty of Medicine, Ankara Yildirim Beyazit University, 06800 Ankara, Türkiye; 2 Department of Allergy and Clinical Immunology, Ankara Bilkent City Hospital, 06800 Ankara, Türkiye; 3 Department of Allergy and Clinical Immunology, Faculty of Medicine, Dokuz Eylul University, 35340 Izmir, Türkiye

**Keywords:** health literacy, drug hypersensitivity, drug allergy, sociodemographic determinants, allergy and immunology

## Abstract

*Background and Objectives:* Health literacy (HL) is an important determinant of health outcomes, but its assessment in patients with drug hypersensitivity reactions (DHRs) remains largely unexplored. This study aimed to evaluate HL levels and identify their sociodemographic determinants among patients with DHRs. *Materials and Methods:* In this single-center, prospective, cross-sectional study, 155 adult patients with a clinical history strongly suggestive of, or diagnostically confirmed, DHR were enrolled at Ankara Bilkent City Hospital (October–December 2025). HL was assessed using the validated Turkey Health Literacy Scale-32 (THLS-32). The associations between sociodemographic and clinical characteristics and HL categories were analyzed using the chi-square test. Variables with *p* < 0.20 in univariable logistic regression were considered for inclusion in a multivariable binary logistic regression model to identify factors independently associated with inadequate or problematic/limited HL. *Results:* The median total THLS-32 score was 32.81 (range: 17.71–50), and 52.9% of patients had inadequate or problematic/limited HL, with the evaluating-information subdimension most affected. In multivariable analysis, higher educational level was associated with lower odds of inadequate or problematic/limited HL (high school: adjusted odds ratio (OR) 0.26, 95% confidence interval (CI) 0.09–0.79; university: adjusted OR 0.15, 95% CI 0.05–0.43), whereas DHR grade 2–3 was associated with higher odds (adjusted OR 4.61, 95% CI 2.06–10.28). The association with age ≥ 35 years did not reach statistical significance after adjustment (*p* = 0.051). *Conclusions:* Inadequate or problematic/limited HL is common among patients with DHRs, particularly when it comes to appraising health information. It is also independently associated with lower educational attainment and more severe reactions. Assessing HL could be integrated into routine evaluations of these patients to support personalized education and safe drug avoidance. However, given the single-center, cross-sectional design of this study, multicenter longitudinal studies are needed to confirm these associations and evaluate whether HL-targeted interventions improve allergy-related outcomes.

## 1. Introduction

Drug allergies are commonly encountered in clinical practice and constitute a significant subset of adverse drug reactions, involving both immunological and non-immunological mechanisms. These reactions may present across a broad clinical spectrum, ranging from mild cutaneous manifestations to severe and potentially life-threatening drug hypersensitivity reactions (DHRs) [1,2,3,4]. Misdiagnosis or inappropriate labeling of drug allergies remains a major clinical concern, as it can lead to unnecessary drug avoidance, reliance on less effective or broader-spectrum alternatives, and increased healthcare costs. Notably, antibiotic allergy labels are associated with increased use of broad-spectrum agents, thereby contributing to antimicrobial resistance [5,6,7].

Health literacy (HL) is a multidimensional construct defined as the capacity of individuals to obtain, process, understand, and appropriately apply health-related information in making informed health decisions [8,9,10]. HL is increasingly recognized as a key determinant of health [8,11] and an important public health goal [12], as it enables individuals not only to understand health information but also to participate actively in decisions about their own care. Adequate HL allows patients to communicate effectively with healthcare providers, follow treatment instructions, use medications correctly, and engage in preventive and self-management behaviors, thereby contributing to better health outcomes [12]. Conversely, limited HL has been consistently associated with poorer health knowledge, difficulty interpreting medication labels and health messages, inappropriate medication use, more frequent hospitalizations and emergency department visits, and overall worse health outcomes, including higher mortality among older adults [11,12]. In the context of drug allergies, inadequate HL may result in patients’ inability to accurately report their allergy history, ineffective communication with healthcare providers, and inadvertent re-exposure to culprit medications [2,8]. On the other hand, adequate HL may help patients understand the nature of their reaction, recognize culprit and potentially cross-reactive drugs, and safely follow avoidance or delabeling recommendations.

A critical challenge in clinical practice is that patients frequently misinterpret adverse drug reactions, often conflating true drug allergies with side effects or drug intolerance. This misunderstanding contributes to the over-labeling of drug allergies, which in turn restricts therapeutic options and complicates clinical management. For instance, a substantial proportion of patients with a reported penicillin allergy are found not to be truly allergic upon comprehensive evaluation. This highlights the crucial role of accurate patient education and appropriate risk stratification [7,13,14].

While the relationship between HL and chronic disease management or medication adherence has been widely investigated, there is a notable lack of data specifically addressing HL among patients with drug allergies [15]. The level of HL and its determinants in this patient group have not been sufficiently elucidated. There is a need for research in this area, particularly regarding patient safety, proper medication use, and the prevention of unnecessary medication use.

Therefore, the aim of this study was to evaluate the level of HL among patients with a history or diagnosis of drug allergy and to identify the sociodemographic and clinical factors associated with HL. The findings are expected to inform targeted patient education strategies in allergy practice.

## 2. Materials and Methods

### 2.1. Study Design, Setting, and Participants

This single-center, prospective, cross-sectional study was designed to evaluate HL levels and their determinants among patients with DHRs using the validated Turkey Health Literacy Scale-32 (THLS-32) [10]. The study was conducted at the outpatient clinics of the Department of Allergy and Clinical Immunology and at the day-care unit where drug provocation tests are performed, at Ankara Bilkent City Hospital, a tertiary referral center in Ankara, Turkey. Patients were recruited consecutively over a two-month period from 16 October to 16 December 2025.

Adult patients (≥18 years) were eligible for inclusion if they had either a clinical history strongly suggestive of DHR or a DHR confirmed through diagnostic allergological evaluation (skin testing, in vitro testing, and/or drug provocation testing), in accordance with the International Consensus on drug allergy [16] and the European Academy of Allergy and Clinical Immunology (EAACI)/European Network on Drug Allergy (ENDA) position paper on drug provocation testing [14]. Patients with a self-reported drug allergy label lacking a corroborating clinical history or diagnostic confirmation were therefore not considered eligible. Patients classified as high-risk based on their clinical history underwent testing with alternative drugs to identify safe treatment options, while diagnostic tests were performed directly in patients classified as low-risk. All eligible patients were interviewed face-to-face by trained study personnel to complete the questionnaire after providing written informed consent.

Eligible patients were excluded if they had cognitive impairment or were unable to understand the questionnaire, or if they declined to participate or did not provide written informed consent.

### 2.2. Ethical Approval

The study protocol was approved by the Ankara Bilkent City Hospital Ethics Committee (approval no. TABED-2-25-1573). All procedures were conducted in accordance with the principles of the Declaration of Helsinki [17]. Written informed consent was obtained from all patients prior to their enrollment in the study.

### 2.3. Data Collection

Data were collected through a structured, interviewer-administered questionnaire conducted face-to-face with patients at the time of their clinical visit. This format was chosen over patient self-completion to minimize missing or incomplete responses, to accommodate patients with limited literacy or visual impairment, and to allow real-time clarification of ambiguous items, thereby ensuring standardized and complete data collection across all participants. The questionnaire comprised two main components: (1) a sociodemographic and clinical characteristics form, including age, sex, educational level, marital status, employment status, income status, and history of drug allergy (e.g., culprit drug group and severity of DHRs); and (2) assessment of HL using the THLS-32 scale [10,18].

### 2.4. Measurement Instrument

The THLS-32 was developed by Okyay et al. in 2016 [10], adapted from the European Health Literacy Survey (HLS-EU) questionnaire [19], and subsequently validated and published by the Turkish Ministry of Health as a reliable instrument for assessing HL in Turkish-speaking populations [10]. The scale assesses HL across two principal domains: treatment/services and disease prevention/health promotion. In addition, it evaluates four core competencies related to health information processing: accessing, understanding, evaluating, and applying information [10,18]. Permission to use the THLS-32 scale for the purposes of this study was obtained from the original scale developers via e-mail correspondence.

The instrument comprises 32 items, each rated on a 5-point Likert-type scale ranging from 1 (very easy) to 4 (very difficult), with an additional response option of 5 (I don’t know). For scoring purposes, responses are recoded such that higher values reflect greater HL proficiency. The overall index score is standardized to a range of 0–50 using the following formula: Index = (mean score − 1) × (50/3). Based on the calculated index score, HL is categorized into four levels: inadequate (0–25), problematic/limited (>25–33), adequate (>33–42), and excellent (>42–50). Individuals classified in the inadequate category experience substantial difficulties in accessing, understanding, evaluating, and applying health-related information. Those in the problematic/limited category possess foundational skills but may encounter challenges when managing more complex health information. Patients in the adequate category generally demonstrate a sufficient level of competency in handling health-related information, whereas those in the excellent category exhibit a high degree of proficiency across all HL domains.

In the present study, THLS-32 scores were computed as a general index, as well as domain-specific indices for treatment/services and disease prevention/health promotion. Furthermore, four subdimension indices were derived: access to information, understanding of information, evaluating information, and applying/using information, all calculated using the same standardization formula. Each index score was subsequently classified according to the predefined HL category thresholds [10,18,20].

### 2.5. Statistical Analysis

All consecutive eligible patients presenting during the two-month study period were enrolled. Sample size adequacy was assessed for the multivariable binary logistic regression using the method of Hsieh, Bloch, and Larsen [21], as implemented in G*Power software (version 3.1.9.7; Heinrich-Heine-Universität Düsseldorf, Düsseldorf, Germany). Because all predictors in the final model were categorical, the calculation was performed for a binary predictor (*X* distribution: binomial, *π* = 0.50), assuming an odds ratio of 3.47, corresponding to a medium effect size according to Chen et al. [22], a two-tailed *α* of 0.05, a statistical power of 0.85, a baseline event probability of 0.50, and *R^2^* = 0.20 for the association of the predictor with other covariates. Under these assumptions, the minimum required sample size was 137 patients; the achieved sample (*N* = 155), with no missing data, exceeded this requirement. In addition, with 73 patients in the smaller outcome group (adequate/excellent HL) and four estimated parameters in the final model, the events-per-variable (EPV) ratio was 18.3, exceeding the conventionally recommended minimum of 10 [23].

All statistical analyses were performed using IBM SPSS Statistics software (version 25.0; IBM Corp., Armonk, NY, USA). Continuous variables were summarized as mean ± standard deviation (SD) or median (range), as appropriate based on data distribution, while categorical variables were expressed as absolute frequencies and percentages. The normality assumption for continuous variables was evaluated using the Kolmogorov–Smirnov test, supplemented by visual inspection of histograms and quantile–quantile (Q-Q) plots. Associations between categorical variables were assessed using the Pearson chi-square test; Fisher’s exact test (Freeman–Halton extension for *r* × *c* tables) was used instead when more than 20% of cells in a contingency table had an expected count < 5 [24].

Binary logistic regression analysis was performed to identify sociodemographic and clinical factors associated with HL, with HL status dichotomized as inadequate or problematic/limited vs. adequate/excellent (reference). First, univariable logistic regression was performed for each candidate variable (age group, sex, marital status, educational level, employment status, income status, culprit drug, and DHR severity grade according to the Brown classification [25]). Variables with a *p* value < 0.20 in univariable analysis (overall *p* value for variables with more than two categories) were considered for inclusion in the multivariable model [26,27], which was constructed using the Enter method. Multicollinearity among candidate predictors was assessed using Cramér’s *V* [28]. Because age group and marital status were strongly associated (Cramér’s *V* = 0.65), only age group was retained in the multivariable model. To avoid sparse cells and unstable estimates, several categories were combined. Primary and middle school education were merged into a single category in both the chi-square and regression analyses. For the logistic regression analyses, age was dichotomized as 18–34 vs. ≥35 years (because only 13 patients were aged ≥ 65 years), income status was dichotomized as income < expenses vs. income ≥ expenses, and DHR grades 2 and 3 were combined and compared with grade 1. Categorical variables were entered using indicator coding, with the first category of each variable (age 18–34 years, primary/middle school education, and DHR grade 1) designated as the reference. Results are presented as crude and adjusted odds ratios (OR) with 95% confidence intervals (CI). Model performance was assessed using the omnibus test of model coefficients, Nagelkerke *R^2^* [29], and the Hosmer–Lemeshow goodness-of-fit test [30]. For all analyses, a two-tailed *p* value < 0.05 was considered statistically significant.

## 3. Results

### 3.1. Sociodemographic Characteristics of the Study Population

A total of 155 patients were enrolled in the study. The sociodemographic and clinical characteristics of the patients are presented in Table 1. The median age was 42 years (range: 19–79). The cohort comprised 82 male (52.9%) and 73 female (47.1%) patients. The majority of patients were married (*n* = 94; 60.6%) and employed (*n* = 94; 60.6%). Regarding educational attainment, the largest proportion of patients held a university degree or higher (*n* = 71; 45.8%), followed by high school graduates *(n* = 42; 27.1%). In terms of occupation, civil servants constituted the most common group (*n* = 42; 27.1%), followed by housewives (*n* = 33; 21.3%) and self-employed patients (*n* = 28; 18.1%). All patients were covered by social security (*n* = 155; 100%). With respect to income status, 41.3% of patients reported that their income was less than their expenses, and an equal proportion reported that income and expenses were balanced, while 17.4% indicated a surplus income.

### 3.2. Clinical Characteristics and Severity of DHRs

With respect to clinical characteristics, the median duration of known drug allergy among patients was 3 years (range: 1–25). When the culprit drug groups were evaluated, analgesics—predominantly non-steroidal anti-inflammatory drugs (NSAIDs)—were the most frequently implicated agents (*n* = 76; 49.0%), followed by antibiotics (*n* = 57; 36.8%) and other drug groups (*n* = 22; 14.2%). The clinical severity of DHRs was graded according to the Brown classification [25]. Based on this grading system, Grade 2 reactions were the most commonly observed (*n* = 63; 40.6%), followed by Grade 1 reactions (*n* = 55; 35.5%) and Grade 3 reactions (*n* = 37; 23.9%), indicating that the majority of patients experienced mild-to-moderate DHR at the time of initial presentation.

### 3.3. Total THLS-32 Health Literacy Scores of the Study Population

The median total THLS-32 score of the patients was 32.81 (range: 17.71–50). When the overall HL category distribution was examined, 40 patients (25.8%) were classified in the “inadequate” category, 42 patients (27.1%) in the “problematic/limited” category, 33 patients (21.3%) in the “adequate” category, and 40 patients (25.8%) in the “excellent” category, indicating that more than half of the patients demonstrated insufficient HL.

When the two main dimensions of the THLS-32 were evaluated separately, the median score for the treatment and services dimension was 31.25 (range: 18.75–50), with the largest proportion of patients classified in the “problematic/limited” category (*n* = 43; 27.7%), followed by the “adequate” (*n* = 40; 25.8%), “inadequate” (*n* = 39; 25.2%), and “excellent” (*n* = 33; 21.3%) categories. Within this dimension, the subdimension with the highest rate of “inadequate” classification was evaluating information (*n* = 61; 39.4%), whereas the using/applying information subdimension demonstrated the highest proportion of patients in the “adequate” category (*n* = 75; 48.4%). Notably, the accessing information subdimension yielded the highest median score within this domain (37.50; range: 16.67–50), while evaluating information recorded the lowest (29.17; range: 12.50–50) (Supplementary Table S1).

For the disease prevention/health promotion dimension, the median score was 31.11 (range: 14.58–50), with the highest proportion of patients classified as “excellent” (*n* = 45; 29.0%), followed by “inadequate” (*n* = 40; 25.8%), “problematic/limited” (*n* = 38; 24.5%), and “adequate” (*n* = 32; 20.6%) categories. Among the subdimensions, access to information exhibited the highest rate of “inadequate” classification (*n* = 65; 41.9%), despite a comparatively higher median score within this domain (33.33; range: 8.33–50). In contrast, the evaluating information and using/applying information subdimensions recorded the lowest median scores in this domain (29.17; range 4.17–50 and 12.50–50, respectively), with using/applying information additionally showing the highest proportion of patients in the “problematic/limited” category (*n* = 48; 31.0%).

**Figure 1:**
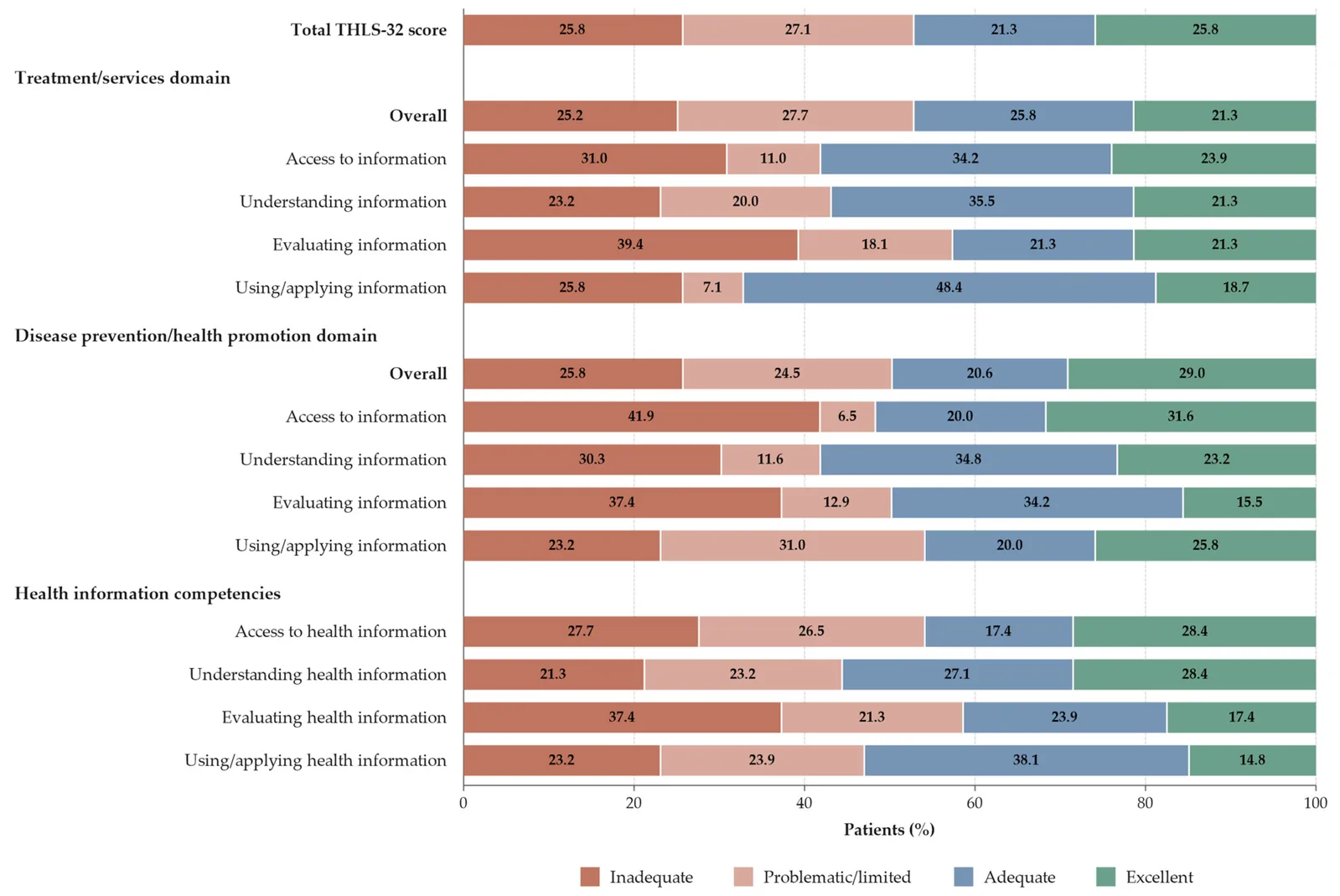
Distribution of health literacy categories across THLS-32 domains and subdimensions (*N* = 155). Bars represent the percentage of patients classified as inadequate, problematic/limited, adequate, or excellent for the total THLS-32 score, the two principal domains (treatment/services; disease prevention/health promotion) with their respective subdimensions, and the four cross-cutting competency domains (access to, understanding, evaluating, and using/applying health information). THLS-32, Turkey Health Literacy Scale-32.

With respect to the four cross-cutting competency domains, the median scores ranged from 30.95 to 35.42. The using/applying health information domain yielded the highest median score (35.42; range: 16.67–50), with the largest proportion of patients classified in the “adequate” category (*n* = 59; 38.1%). Conversely, the evaluating health information domain recorded the lowest median score (30.95; range: 14.58–50) and the highest proportion of patients in the “inadequate” category (*n* = 58; 37.4%). The access to health information and understanding health information domains showed similar distributions, with approximately one quarter of patients achieving “excellent” HL in each domain (*n* = 44; 28.4% and *n* = 44; 28.4%, respectively). The distribution of HL scores and categories across THLS-32 dimensions is presented in Figure 1.

Overall, these findings indicate that HL levels among patients with DHRs were predominantly concentrated in the lower categories, with the lowest median scores observed for the evaluating information subdimensions across both main dimensions, as well as in the evaluating health information competency domain.

### 3.4. Association of Sociodemographic Characteristics with HL Categories

A statistically significant association was observed between sex and HL levels (*p* < 0.001). Male patients were more frequently classified in the “excellent” category (*n* = 30; 36.6%), whereas female patients were predominantly distributed across the “problematic/limited” (*n* = 27; 37.0%) and “adequate” (*n* = 21; 28.8%) categories, indicating a more polarized HL distribution among male patients (Table 2).

With respect to age, a statistically significant association was identified between age group and HL levels (*p* < 0.001). Patients aged 18–34 years demonstrated the highest proportion of “excellent” HL (*n* = 26; 40.0%), while those aged 35–64 years were predominantly concentrated in the “problematic/limited” (*n* = 26; 33.8%) and “inadequate” (*n* = 25; 32.5%) categories. Among patients aged 65 years and older, no patients were classified in the “excellent” category, and the “inadequate” category was predominant (*n* = 8; 61.5%), suggesting a clear inverse relationship between advancing age and HL level.

Marital status was also significantly associated with HL distribution (*p* < 0.001). Single patients demonstrated a markedly more favorable HL profile, with more than half classified in the “excellent” category (*n* = 33; 54.1%). In contrast, married patients were predominantly distributed across the “inadequate” (*n* = 29; 30.9%) and “problematic/limited” (*n* = 30; 31.9%) categories, with only a small proportion achieving “excellent” HL (*n* = 7; 7.4%).

Regarding educational attainment, a statistically significant association was observed between educational level and HL categories (*p* < 0.001). Patients with primary or middle school education were predominantly classified in the “inadequate” category (*n* = 21; 50.0%), and none of the high school graduates achieved “excellent” HL. In contrast, patients with a university degree or higher demonstrated the most favorable distribution, with the majority classified in the “excellent” category (*n* = 37; 52.1%) and only a small proportion in the “inadequate” category (*n* = 4; 5.6%), indicating a strong positive association between higher educational attainment and better HL outcomes.

No statistically significant association was identified between employment status and HL categories (*p* = 0.181). Although a higher proportion of employed patients achieved “excellent” HL (30.9% vs. 18.0%), the overall distribution of HL levels did not differ significantly between employed and unemployed patients.

Finally, income status was significantly associated with HL levels (*p* < 0.001). Among patients whose income exceeded their expenses, the majority were classified in the “excellent” category (*n* = 15; 55.6%), and none were classified as “inadequate.” Conversely, patients whose income was less than their expenses were predominantly distributed in the “inadequate” (*n* = 22; 34.4%) and “adequate” (*n* = 17; 26.6%) categories. Patients with balanced income and expenses showed the highest concentration in the “problematic/limited” category (*n* = 28; 43.8%), further underscoring the positive relationship between financial status and HL. The full distribution of HL categories across all sociodemographic variables is presented in Table 2.

### 3.5. Factors Associated with Inadequate or Problematic/Limited Health Literacy

The results of the univariable and multivariable logistic regression analyses are presented in Table 3. In univariable analysis, patients aged ≥ 35 years had higher odds of inadequate or problematic/limited HL than those aged 18–34 years (OR 3.48, 95% CI 1.78–6.78, *p* < 0.001), whereas single patients had lower odds than married patients (OR 0.36, 95% CI 0.18–0.70, *p* = 0.003). Educational level was strongly associated with HL (overall *p* < 0.001); compared with patients with primary/middle school education, the odds of inadequate or problematic/limited HL were lower among high school graduates (OR 0.22, 95% CI 0.08–0.61, *p* = 0.003) and university graduates (OR 0.11, 95% CI 0.04–0.28, *p* < 0.001). Patients with DHR grade 2–3 had higher odds of inadequate or problematic/limited HL than those with grade 1 (OR 3.65, 95% CI 1.82–7.33, *p* < 0.001). Sex (*p* = 0.276), employment status (*p* = 0.569), income status (*p* = 0.779), and culprit drug (overall *p* = 0.310) were not associated with HL.

In the multivariable model including age group, educational level, and DHR grade, educational level and DHR grade remained independently associated with inadequate or problematic/limited HL. Compared with primary/middle school education, high school education (adjusted OR 0.26, 95% CI 0.09–0.79, *p* = 0.017) and university education (adjusted OR 0.15, 95% CI 0.05–0.43, *p* < 0.001) were associated with lower odds of inadequate or problematic/limited HL. DHR grade 2–3 was associated with an approximately 4.6-fold higher odds of inadequate or problematic/limited HL compared with grade 1 (adjusted OR 4.61, 95% CI 2.06–10.28, *p* < 0.001). The association with age ≥ 35 years did not reach statistical significance after adjustment (adjusted OR 2.25, 95% CI 0.99–5.07, *p* = 0.051). Marital status was not included in the multivariable model because of its strong association with age (Cramér’s *V* = 0.65). The overall model was statistically significant (omnibus *χ*^2^ = 44.37, *df* = 4, *p* < 0.001; Nagelkerke *R*^2^ = 0.332), although the Hosmer–Lemeshow test indicated some lack of fit (χ^2^ = 16.72, *df* = 7, *p* = 0.019).

## 4. Discussion

This cross-sectional study investigated HL levels among patients with DHRs attending an outpatient allergy and immunology clinic, and examined the sociodemographic determinants of HL using the THLS-32 instrument [10]. To our knowledge, this is the first study to specifically assess HL in a population of patients with DHRs using a validated, multidimensional HL scale. The existing literature on HL in allergy and immunology settings remains remarkably sparse, and no prior study has directly measured HL levels among patients with drug allergies using the THLS-32 or any equivalent comprehensive HL instrument [31]. The present study directly addresses this gap, and its findings carry important clinical and public health implications for the management of DHRs.

The median total THLS-32 score in the present study was 32.81 (range: 17.71–50), placing the overall sample within the “problematic/limited” HL category. Furthermore, more than half of the patients (52.9%) were classified in either the “inadequate” or “problematic/limited” categories, indicating that a substantial proportion of this population lacks the HL competencies necessary to effectively navigate health-related information. The pattern of sociodemographic determinants of HL observed in the present study is broadly consistent with that reported in the general Turkish population. Notably, a large representative national survey of the Turkish adult population (*n* = 4924) [32] using an HLS-EU-based instrument reported a general HL index of 30.4 and classified approximately two-thirds (64.6%) of participants as having inadequate or problematic/limited HL, figures that are numerically close to—and marginally less favorable than—those observed in our DHR sample (median 32.81; 52.9% inadequate or problematic/limited), suggesting that HL in this clinical population broadly mirrors, rather than falls substantially below, that of the general Turkish population. Differences in the specific instruments used (HLS-EU-based vs. THLS-32) and in sampling methodology nonetheless warrant cautious interpretation of this comparison.

A large-scale national study utilizing the THLS-32 in 6228 individuals [33] demonstrated that HL scores were inversely associated with outpatient and emergency department admissions, and that sex, age, income, and educational level were also associated with healthcare utilization. In the present study, male sex (excellent HL: 36.6% vs. 13.7% in females), younger age (excellent HL: 40.0% among patients aged 18–34 years vs. 0% and predominantly inadequate HL [61.5%] among those aged ≥ 65 years), higher educational attainment (excellent HL: 52.1% among university graduates vs. 50.0% inadequate HL among primary/middle-school graduates), and higher income (excellent HL: 55.6% when income exceeded expenses vs. 0% inadequate HL in this group) were each associated with more favorable HL classification (all *p* < 0.001 in the chi-square analysis), although, of these factors, only educational attainment remained independently associated with HL in the multivariable model. In line with these findings, a cross-sectional study comparing public and private hospital outpatients in Turkey [34] found that age, education, sex, and income were significant predictors of adequate HL across both settings. Future studies directly comparing DHR and general-population samples using the same instrument would help clarify whether HL is disproportionately impaired in this clinical population.

The most directly comparable study to the present work is a recent cross-sectional investigation of HL in patients with inborn errors of immunity (IEI)—another clinically complex immunological condition—conducted at an allergy and immunology department in Turkey using the THLS-32 [20]. That study reported that 44.4% of IEI patients had inadequate or problematic HL, and identified age, sex, education, and marital status as significant determinants, findings that are strikingly consistent with those of the present study. Notably, the present DHR cohort demonstrated a similarly substantial burden of insufficient HL (52.9%); although both figures are numerically somewhat lower than the 64.6% inadequate or problematic/limited rate reported in a nationally representative Turkish general-population survey [32], this comparison suggests that HL deficits are not disproportionately amplified in these allergic/immunological populations relative to the general population, but rather that a consistently substantial proportion of patients—whether in the general population or in these clinical subgroups—lack the HL competencies needed to navigate complex health information. Given the particular complexity of information that patients with allergic and immunological conditions must process, this shared burden of insufficient HL nonetheless warrants targeted clinical attention across the spectrum of these conditions, rather than being specific to any single diagnosis. The relevance of HL in the context of DHRs is particularly compelling, as patients with drug allergies are frequently required to engage with complex medical information—including allergen avoidance, alternative drug selection, and the interpretation of drug provocation test results—tasks that demand a high degree of HL proficiency. Indeed, inadequate documentation and communication between healthcare providers, as well as limited HL and knowledge among patients, have been suggested to contribute to the recurrence of allergic reactions, highlighting the potential role of HL in preventing avoidable drug-related harm [35].

When the two principal THLS-32 domains were examined separately, both the treatment/services and disease prevention/health promotion dimensions yielded median scores in the “problematic/limited” range (31.25 and 31.11, respectively). Overall, the evaluating information competency emerged as the most deficient area: it recorded the highest rate of “inadequate” classification in the treatment/services domain (*n* = 61; 39.4%) and in the cross-cutting competency domains (*n* = 58; 37.4%), and the second-highest in the disease prevention/health promotion domain (*n* = 58; 37.4%), after access to information (n = 65; 41.9%). This pattern is broadly consistent with prior evidence suggesting that the appraisal and critical evaluation of health information is often the most cognitively demanding HL competency, and can remain impaired even when other competencies are relatively preserved [36].

The clinical implications of this finding are considerable; patients who are unable to critically appraise health information may be more susceptible to misinformation, less equipped to make informed decisions regarding drug exposure, and less likely to engage productively in shared decision-making with their healthcare providers. In the context of penicillin and NSAID allergy—the most prevalent culprit drug groups in this cohort—poor evaluative HL may contribute to the perpetuation of inaccurate allergy labels, unnecessary avoidance of first-line medications, and suboptimal antibiotic stewardship outcomes [37,38].

Although all four core HL competencies contribute to safe drug allergy management, they are not likely to carry equal clinical weight in this context. Accessing information is relevant, for example, when a patient must locate their own allergy documentation or identify which drugs belong to a culprit group before a new prescription is issued. Understanding information is required to correctly interpret a drug provocation test result or an allergist’s explanation of cross-reactivity between antibiotics. Applying information involves translating this knowledge into practice—for instance, correctly avoiding a labeled culprit drug or its cross-reactive alternatives when presenting to a new prescriber. Evaluating information, however, may be the most safety-critical of the four in this population, as it underlies a patient’s ability to critically appraise conflicting or unreliable sources (e.g., online forums or anecdotal reports) about which drugs are “safe,” to distinguish a true immune-mediated drug allergy from intolerance or a coincidental reaction, and to judge when a previously avoided drug might in fact be suitable following formal evaluation. Given that our cohort demonstrated a comparatively preserved using/applying competency (48.4% “adequate” in the treatment/services domain) alongside a disproportionately impaired evaluating competency, interventions that specifically target this evaluative skill—rather than general information provision—may offer the greatest benefit for reducing diagnostic mislabeling and inappropriate drug avoidance in patients with DHRs.

With regard to the cross-cutting competency domains, the evaluating health information domain recorded the lowest median score (30.95) and the highest rate of “inadequate” classification (37.4%), further corroborating the pattern of evaluative deficiency observed across the THLS-32 subdimensions. Conversely, the using/applying health information domain yielded the highest median score (35.42), with the largest proportion of patients classified in the “adequate” category. These findings collectively suggest that interventions specifically targeting critical health information appraisal skills—rather than general information provision alone—may be of particular value in this patient population.

The present study identified several sociodemographic factors associated with HL. The distribution of HL categories differed significantly between sexes (*p* < 0.001), with male patients more frequently achieving “excellent” HL (36.6% vs. 13.7%), in line with previous reports of sex-related differences in HL [39]. However, male patients were also more frequently classified in the “inadequate” category (30.5% vs. 20.5%), and sex was not associated with inadequate or problematic/limited HL in the logistic regression analysis (OR 1.42, 95% CI 0.75–2.68, *p* = 0.276). Thus, the sex difference observed in our cohort reflected a more polarized HL distribution among male patients rather than consistently lower HL among female patients. Sex-related differences in HL have been documented in multiple national and international studies and may reflect differential access to education, occupational exposure to health-related information, and sociocultural patterns of health information-seeking behavior [8,40]. In the context of drug allergy management, these findings suggest that sex alone is not a sufficient criterion for identifying patients at risk of limited HL, and that HL-sensitive communication should be guided by individual assessment rather than by sex.

A clear and statistically significant inverse relationship was observed between age and HL levels. While patients aged 18–34 years most frequently achieved “excellent” HL (40.0%), those aged 65 years and older were predominantly classified in the “inadequate” category (61.5%), with no patients in this age group achieving “excellent” HL. It should be noted, however, that the ≥65-year age group comprised a relatively small subsample (n = 13) in this cohort; therefore, this finding should be interpreted with caution and requires confirmation in larger, adequately powered samples of older patients with DHRs. In the logistic regression analysis, patients aged ≥ 35 years had higher odds of inadequate or problematic/limited HL than those aged 18–34 years (OR 3.48, 95% CI 1.78–6.78); however, this association was attenuated and did not reach statistical significance after adjustment for educational level and DHR grade (adjusted OR 2.25, 95% CI 0.99–5.07, *p* = 0.051), suggesting that part of the age-related HL disadvantage may be explained by lower educational attainment among older patients. This age-related decline in HL is well documented in the Turkish literature and has been attributed to lower educational attainment in older cohorts, reduced exposure to digital health information, declining cognitive processing capacity, and greater comorbidity burden [41,42,43]. In older patients with DHRs, these HL deficiencies may be compounded by polypharmacy, substantially increasing the complexity of drug allergy management and the potential consequences of misunderstanding medication-related information.

In the bivariate analysis, marital status was significantly associated with HL distribution, with single patients demonstrating markedly higher HL levels than married patients. More than half of single patients were classified in the “excellent” category (54.1%), compared to only 7.4% of married patients. This finding is consistent with that of the aforementioned IEI study, which similarly reported lower HL among married patients compared to single patients using the THLS-32 [20]. As noted in that study, this association likely reflects the confounding influence of age and educational level, as single patients in clinical populations tend to be younger and more highly educated. Consistent with this interpretation, marital status was strongly associated with age group in our cohort (Cramér’s *V* = 0.65); therefore, it was not entered into the multivariable model to avoid collinearity, and its crude association with HL is likely to largely reflect the effect of age rather than marital status per se.

Educational attainment emerged as one of the strongest determinants of HL in this study. Patients with a university degree or higher were most frequently classified in the “excellent” category (52.1%), whereas half of those with primary or middle school education were classified as “inadequate” (50.0%). This strong positive gradient between educational level and HL is one of the most consistently replicated findings in the HL literature globally, and underscores the foundational role of general literacy and numeracy skills in enabling individuals to access, understand, and apply health information [44]. Notably, educational level remained independently associated with HL in the multivariable analysis, with high school graduates (adjusted OR 0.26, 95% CI 0.09–0.79) and university graduates (adjusted OR 0.15, 95% CI 0.05–0.43) having substantially lower odds of inadequate or problematic/limited HL than patients with primary/middle school education. In the specific context of drug allergy, patients with lower educational attainment may be particularly disadvantaged in understanding written discharge instructions, drug allergy cards, and medication labeling—all of which are integral to safe drug allergy management [35,45].

No statistically significant association was identified between employment status and HL, either in the four-category analysis (*p* = 0.181) or in the univariable logistic regression analysis (OR 0.83, 95% CI 0.43–1.58, *p* = 0.569); therefore, employment status was not included in the multivariable model. The broadly homogeneous distribution of HL levels between employed and unemployed patients suggests that occupational status per se is not a major determinant of HL in this population, although this finding may also reflect the heterogeneity of the unemployed group, which included housewives, retired patients, and students.

Income status was significantly associated with HL levels. A higher proportion of patients whose income exceeded their expenses achieved excellent HL (55.6%), whereas no participants in this group were classified as having inadequate HL. However, the association between income status and HL was not observed when HL was dichotomized as inadequate or problematic/limited vs. adequate/excellent (income ≥ expenses vs. income < expenses, OR 1.10, 95% CI 0.58–2.08, *p* = 0.779), indicating that the income gradient was mainly reflected in the likelihood of achieving excellent HL rather than in the proportion of patients with insufficient HL. The observed association between income status and HL is consistent with evidence suggesting that financial resources may influence access to education, health services, and health-promoting resources [46]. In the context of DHRs, limited financial resources may also pose barriers to attending specialist allergy clinics, completing drug provocation tests, or accessing safer alternative medications following a confirmed drug allergy diagnosis [46,47,48].

A novel finding of this study was the independent association between DHR severity and HL. Patients with grade 2–3 reactions had more than fourfold higher odds of inadequate or problematic/limited HL than those with grade 1 reactions, even after adjustment for age and educational level (adjusted OR 4.61, 95% CI 2.06–10.28). Because of the cross-sectional design, the direction of this association cannot be determined. Lower HL may contribute to more severe reactions through delayed recognition of early symptoms, inadequate communication of previous reactions to healthcare providers, or inadvertent re-exposure to culprit or cross-reactive drugs [35]. Alternatively, patients with more severe reactions may differ in other unmeasured characteristics, such as comorbidities or patterns of healthcare use, that are related to HL. Regardless of its direction, this finding suggests that patients with more severe DHRs, who face the greatest risk from re-exposure, may be those least equipped to understand and apply drug allergy information, and may therefore benefit most from HL-sensitive education and structured written avoidance instructions.

Although its cross-sectional design captures associations rather than effects on long-term outcomes, this study has several practical implications for tertiary allergy clinics. First, brief HL screening at the initial visit, using validated single-item questions [49], could identify patients who need adapted communication, particularly patients with lower educational attainment and those with more severe (grade 2–3) reactions. Second, because the “evaluating information” competency was the most impaired in our cohort, education should not stop at telling patients which drugs to avoid. It should help them understand why a reaction does or does not represent true hypersensitivity, and teach-back should be used to confirm understanding [50]. Third, consent for drug provocation testing and delabeling could be improved with plain-language materials and visual explanations of the procedure and its risks. After delabeling, patients should receive a structured written summary, and their health records should be updated, to prevent re-labeling [37,38]. Finally, written drug allergy action plans or allergy passports should be adapted to HL level. They should list culprit drugs, cross-reactive agents, and safe alternatives in simple language, with pictograms where appropriate [35,45,51,52]. Prospective studies should assess whether such HL-tailored interventions improve label accuracy, delabeling uptake, and avoidance of accidental re-exposure. The main components of DHR management, the HL competencies they require, the potential consequences of limited HL, and corresponding HL-sensitive strategies are summarized in Supplementary Table S2.

Several limitations of this study should be acknowledged. First, the cross-sectional design precludes causal inference regarding the observed associations between sociodemographic factors and HL levels. Second, the single-center recruitment setting may limit the generalizability of findings to broader DHR populations attending other primary or tertiary care settings. Third, social desirability bias cannot be entirely excluded given the interviewer-administered nature of the questionnaire. Fourth, although the sample size was adequate for the final multivariable model (EPV = 18.3), the Hosmer–Lemeshow test suggested some lack of calibration (χ^2^ = 16.72, *df* = 7, *p* = 0.019), possibly related to the small number of covariate patterns; therefore, the adjusted estimates should be interpreted with caution. In addition, the dichotomization of HL and the collapsing of some categories (e.g., age group and DHR grade) may have resulted in some loss of information. Fifth, the small number of patients aged ≥ 65 years (*n* = 13) limits the precision and generalizability of the observed association between advanced age and inadequate HL, and this finding should be validated in future studies with larger elderly subgroups. Sixth, the study did not evaluate the association between HL and clinically relevant DHR outcomes, such as confirmed drug allergy, delabeling, or completion of the diagnostic work-up. Future prospective studies should investigate whether HL influences these outcomes and affects patients’ engagement with allergy care. Finally, longitudinal studies are needed to establish causal pathways and identify the most influential determinants of HL among patients evaluated for DHRs. The development and evaluation of targeted HL interventions—including simplified patient education materials, pharmacist-led counseling, and digital health tools tailored to different HL levels—represent promising directions for future research in this population [35,51,53].

**Figure 2:**
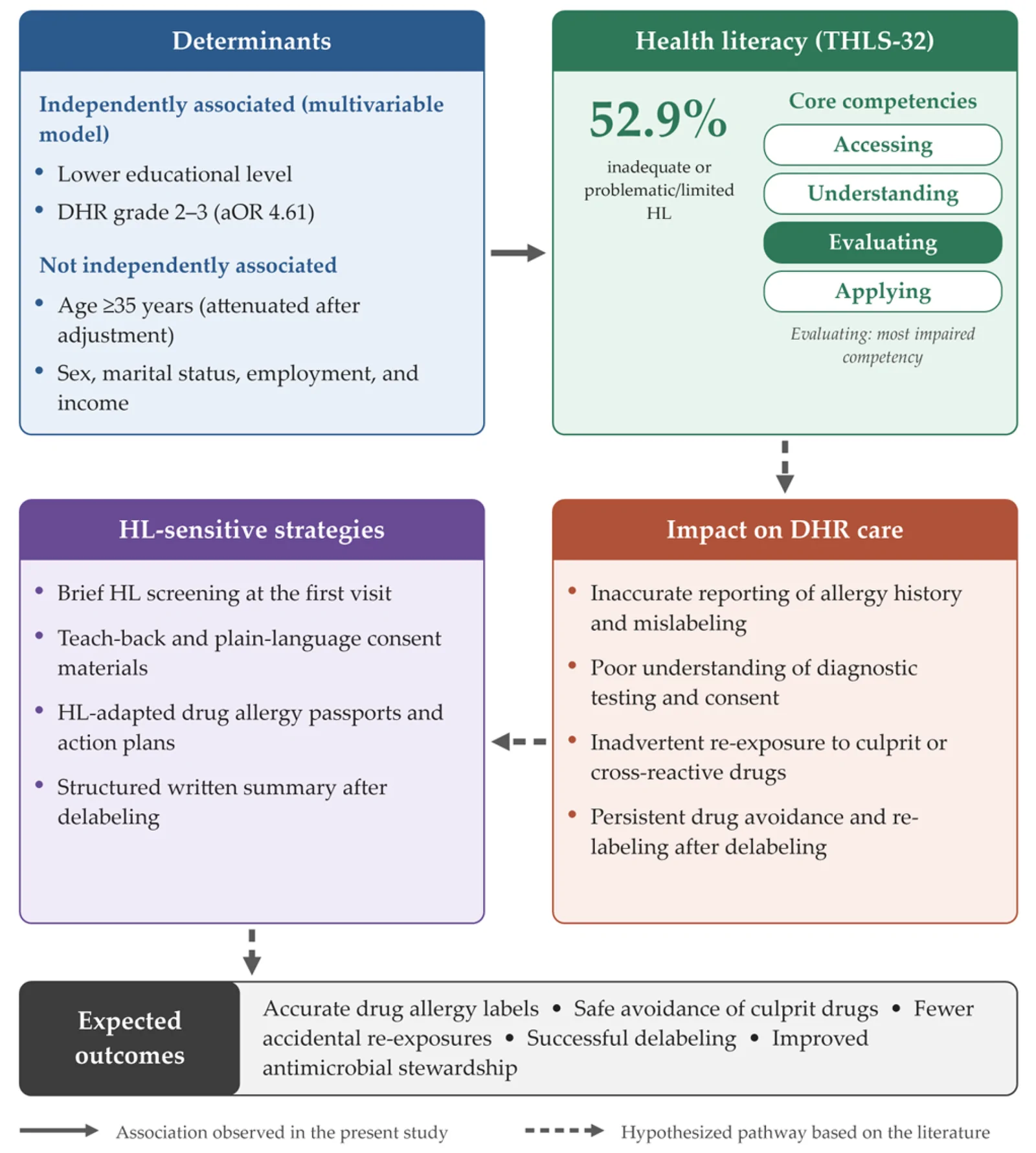
Conceptual framework of health literacy in patients with drug hypersensitivity reactions. The framework integrates the determinants of HL identified in the present study, the distribution of HL and its core competencies, the potential impact of limited HL on DHR care, and HL-sensitive strategies with their expected outcomes. Solid arrow: association observed in the present study (cross-sectional design; the direction of the association cannot be inferred). Dashed arrows: hypothesized pathways based on the literature. DHR, drug hypersensitivity reaction; HL, health literacy; THLS-32, Turkey Health Literacy Scale-32; aOR, adjusted odds ratio.

A conceptual overview integrating the determinants of HL identified in this study, the potential impact of limited HL on DHR care, and HL-sensitive strategies is presented in Figure 2.

## 5. Conclusions

In conclusion, this study demonstrates that a substantial proportion of patients with DHRs exhibit inadequate or problematic/limited HL, with particular and consistent deficiencies in the critical evaluation of health information across most THLS-32 domains. The findings highlight that this patient population shares the HL burden observed in other immunological conditions, yet has remained largely unstudied in the HL literature to date. In univariable analyses, older age, marital status, lower educational attainment, and more severe DHRs were associated with inadequate or problematic/limited HL. In the multivariable model, lower educational attainment and grade 2–3 DHRs remained independently associated with higher odds of inadequate or problematic/limited HL, whereas the association with age was attenuated after adjustment. These findings underscore the need for HL-sensitive clinical communication strategies, individualized patient education, and routine HL screening in allergy and immunology practice, particularly for patients with lower educational attainment and those with more severe DHRs. Multicenter longitudinal studies are needed to clarify the direction of these associations and to evaluate whether HL-targeted interventions improve outcomes in this population.

## Figures and Tables

**Table 1:** Sociodemographic and clinical characteristics of patients (*N* = 155).

Variable	Category	*n* (%)/Median (Range)
**Age (years)**		42 (19–79)
**Sex**		
	Male	82 (52.9)
	Female	73 (47.1)
**Marital status**		
	Married	94 (60.6)
	Single	61 (39.4)
**Educational level**		
	Primary school	17 (11.0)
	Middle school	25 (16.1)
	High school	42 (27.1)
	University degree or higher	71 (45.8)
**Employment status**		
	Employed	94 (60.6)
	Unemployed	61 (39.4)
**Occupation**		
	Civil servant	42 (27.1)
	Housewife	33 (21.3)
	Self-employed	28 (18.1)
	Retired	24 (15.5)
	Worker	21 (13.5)
	Student	4 (2.6)
	Tradesperson	3 (1.9)
**Social security**		
	Covered	155 (100)
**Income status**		
	Income < expenses	64 (41.3)
	Income = expenses	64 (41.3)
	Income > expenses	27 (17.4)
**Drug allergy duration (years)**		3 (1–25)
**Culprit drug group**		
	Analgesics (NSAIDs predominant)	76 (49.0)
	Antibiotics	57 (36.8)
	Others	22 (14.2)
**Severity of DHR**		
	Grade 1	55 (35.5)
	Grade 2	63 (40.6)
	Grade 3	37 (23.9)

Data are presented as number (percentage) or median (range), as appropriate. DHR, drug hypersensitivity reaction; NSAIDs, non-steroidal anti-inflammatory drugs.

**Table 2:** Association between sociodemographic characteristics and health literacy categories among patients (*N* = 155).

Variable	Category	Inadequate, *n* (%)	Problematic/Limited, *n* (%)	Adequate, *n* (%)	Excellent, *n* (%)	*p* Value
**Sex**	Male	25 (30.5)	15 (18.3)	12 (14.6)	30 (36.6)	**<0.001**
	Female	15 (20.5)	27 (37.0)	21 (28.8)	10 (13.7)	
**Age (years)**	18–34	7 (10.8)	16 (24.6)	16 (24.6)	26 (40.0)	**<0.001 ^a^**
	35–64	25 (32.5)	26 (33.8)	12 (15.6)	14 (18.2)	
	65+	8 (61.5)	0 (0.0)	5 (38.5)	0 (0.0)	
**Marital status**	Single	11 (18.0)	12 (19.7)	5 (8.2)	33 (54.1)	**<0.001**
	Married	29 (30.9)	30 (31.9)	28 (29.8)	7 (7.4)	
**Educational level**	Primary/middle school	21 (50.0)	14 (33.3)	4 (9.5)	3 (7.1)	**<0.001**
	High school	15 (35.7)	7 (16.7)	20 (47.6)	0 (0.0)	
	University degree or higher	4 (5.6)	21 (29.6)	9 (12.7)	37 (52.1)	
**Employment status**	Employed	21 (22.3)	27 (28.7)	17 (18.1)	29 (30.9)	0.181
	Unemployed	19 (31.1)	15 (24.6)	16 (26.2)	11 (18.0)	
**Income status**	Income < expenses	22 (34.4)	11 (17.2)	17 (26.6)	14 (21.9)	**<0.001**
	Income = expenses	18 (28.1)	28 (43.8)	7 (10.9)	11 (17.2)	
	Income > expenses	0 (0.0)	3 (11.1)	9 (33.3)	15 (55.6)	

*p* values were derived from the Pearson chi-square test unless otherwise indicated. Statistically significant results (*p* < 0.05) are indicated in bold. ^a^ Fisher’s exact test (Freeman–Halton extension) was used for age group because more than 20% of cells had an expected count < 5.

**Table 3:** Univariable and multivariable logistic regression analysis of factors associated with inadequate or problematic/limited health literacy.

Variable	Crude OR (95% CI)	*p*	Adjusted OR (95% CI)	*p*
**Age**				
18–34 years	1 (Ref)		1 (Ref)	
≥35 years	3.48 (1.78–6.78)	<0.001	2.25 (0.99–5.07)	0.051
**Sex**				
Male	1 (Ref)		—	
Female	1.42 (0.75–2.68)	0.276	—	
**Marital status**				
Married	1 (Ref)		— ^a^	
Single	0.36 (0.18–0.70)	0.003	— ^a^	
**Education level**		<0.001 ^b^		0.002 ^b^
Primary/middle school	1 (Ref)		1 (Ref)	
High school	0.22 (0.08–0.61)	0.003	0.26 (0.09–0.79)	0.017
University degree or higher	0.11 (0.04–0.28)	<0.001	0.15 (0.05–0.43)	<0.001
**Employment status**				
Unemployed	1 (Ref)		—	
Employed	0.83 (0.43–1.58)	0.569	—	
**Income status**				
Income < expenses	1 (Ref)		—	
Income ≥ expenses	1.10 (0.58–2.08)	0.779	—	
**Culprit drug**		0.310 ^b^		
Analgesic	1 (Ref)		—	
Antibiotic	1.04 (0.52–2.06)	0.920	—	
Other	2.14 (0.79–5.85)	0.137	—	
**DHR grade**				
Grade 1	1 (Ref)		1 (Ref)	
Grade 2–3	3.65 (1.82–7.33)	<0.001	4.61 (2.06–10.28)	<0.001

Variables with *p* < 0.20 in univariable analysis were considered for the multivariable model (enter method). —, not included in the multivariable model. ^a^ Marital status was not included because of collinearity with age (Cramér’s *V* = 0.65). ^b^ Overall *p* value for the variable. Multivariable model: omnibus *χ*^2^ = 44.37, *df* = 4, *p* < 0.001; Nagelkerke *R*^2^ = 0.332; Hosmer–Lemeshow *χ*^2^ = 16.72, *df* = 7, *p* = 0.019. OR, odds ratio; CI, confidence interval; DHR, drug hypersensitivity reaction; Ref, reference category.

## Data Availability

The datasets generated and/or analyzed during the current study are not publicly available due to privacy and ethical restrictions but are available from the corresponding author on reasonable request.

## Supplementary Materials

The following supporting information can be downloaded at: https://www.mdpi.com/article/10.3390/medicina62102024/s1, Table S1: THLS-32 Health literacy scores of study population in matrix components (*N* = 155). Table S2: Overview of the main components of drug hypersensitivity reaction management, the health literacy competencies they require, and the potential impact of limited health literacy.

## Abbreviations

The following abbreviations are used in this manuscript:

DHR	Drug hypersensitivity reaction
EPV	Events per variable
HL	Health literacy
THLS-32	Turkey Health Literacy Scale-32
HLS-EU	European Health Literacy Survey
IEI	Inborn errors of immunity
NSAIDs	Non-steroidal anti-inflammatory drugs
SD	Standard deviation
SPSS	Statistical Package for the Social Sciences
aOR	Adjusted odds ratio
CI	Confidence interval
EAACI	European Academy of Allergy and Clinical Immunology
ENDA	European Network on Drug Allergy
OR	Odds ratio
Q-Q	Quantile–quantile
