# Supplementary material for: Health Literacy Among Patients Evaluated for Drug Hypersensitivity in a Tertiary Allergy Clinic: A Cross-Sectional Study
Source: Medicina (Kaunas). 2026 Oct 19;62(10):2024. doi: 10.3390/medicina62102024 (PMC13707197; doi:10.3390/medicina62102024)
Supplement: Supplementary file 1 [file medicina-62-02024-s001.zip › medicina-4564581-supplementary.pdf]

Supplementary Table S1. THLS-32 Health literacy scores of study population in matrix components (N = 155).

Category / Subdimension	Median (Range)	Health Literacy Category — General Index, n (%)			
		Inadequate	Problematic / Limited	Adequate	Excellent
**Total THLS-32 Score**	32.81 (17.71–50)	40 (25.8)	42 (27.1)	33 (21.3)	40 (25.8)
**Treatment and Services**	31.25 (18.75–50)	39 (25.2)	43 (27.7)	40 (25.8)	33 (21.3)
Access to Information	37.50 (16.67–50)	48 (31.0)	17 (11.0)	53 (34.2)	37 (23.9)
Understanding Information	33.33 (16.67–50)	36 (23.2)	31 (20.0)	55 (35.5)	33 (21.3)
Evaluating Information	29.17 (12.50–50)	61 (39.4)	28 (18.1)	33 (21.3)	33 (21.3)
Using / Applying Information	33.33 (16.67–50)	40 (25.8)	11 (7.1)	75 (48.4)	29 (18.7)
**Disease Prevention / Health Promotion**	31.11 (14.58–50)	40 (25.8)	38 (24.5)	32 (20.6)	45 (29.0)
Access to Information	33.33 (8.33–50)	65 (41.9)	10 (6.5)	31 (20.0)	49 (31.6)
Understanding Information	33.33 (16.67–50)	47 (30.3)	18 (11.6)	54 (34.8)	36 (23.2)
Evaluating Information	29.17 (4.17–50)	58 (37.4)	20 (12.9)	53 (34.2)	24 (15.5)
Using / Applying Information	29.17 (12.50–50)	36 (23.2)	48 (31.0)	31 (20.0)	40 (25.8)
**Access to Health Information**	31.25 (16.67–50)	43 (27.7)	41 (26.5)	27 (17.4)	44 (28.4)
**Understanding Health Information**	33.33 (16.67–50)	33 (21.3)	36 (23.2)	42 (27.1)	44 (28.4)
**Evaluating Health Information**	30.95 (14.58–50)	58 (37.4)	33 (21.3)	37 (23.9)	27 (17.4)
**Using / Applying Health Information**	35.42 (16.67–50)	36 (23.2)	37 (23.9)	59 (38.1)	23 (14.8)

Data are presented as median (range) or number (percentage), as appropriate. THLS-32, Turkey Health Literacy Scale-32.

Supplementary Table S2. Overview of the main components of drug hypersensitivity reaction management, the health literacy competencies they require, and the potential impact of limited health literacy.

Component of DHR management	Current approach	Main HL competency required	Potential impact of limited HL	HL-sensitive strategies
History taking and documentation	Detailed clinical history (culprit drug, timing, symptoms, and severity) and accurate recording of allergy labels [1,16]	Accessing and understanding	Incomplete or inaccurate reporting of the reaction; over-labeling and mislabeling of drug allergy [5,6]	Structured interviews; review of medical records; brief HL screening at the initial visit [49]
Risk stratification and diagnostic testing	Skin tests, in vitro tests, and drug provocation testing selected according to the individual risk profile [14,16]	Understanding and evaluating	Misunderstanding of the purpose and risks of testing; reluctance to consent to diagnostic evaluation	Plain-language consent materials and visual explanations; confirmation of understanding with teach-back [50]
Management of acute reactions	Discontinuation of the culprit drug; antihistamines and corticosteroids; intramuscular epinephrine for anaphylaxis [3,4]	Understanding and applying	Delayed recognition of early symptoms; delayed or incorrect use of emergency treatment	Written emergency action plans; demonstration and teach-back [50]
Avoidance of culprit and cross-reactive drugs	Strict avoidance of the culprit drug; identification of safe alternatives through allergological evaluation [14,16]	Accessing and applying	Inadvertent re-exposure; failure to inform new prescribers about the allergy [35]	Drug allergy cards or passports listing culprit, cross-reactive, and safe drugs in simple language, with pictograms where appropriate [35,45,51,52]
Drug desensitization	Temporary induction of tolerance under specialist supervision when no suitable alternative is available [3]	Understanding and evaluating	Misunderstanding that tolerance is temporary; poor adherence to the protocol	Shared decision-making; clear written instructions on the protocol and its limitations
Delabeling of inaccurate allergy labels	Removal of the allergy label after a negative evaluation and updating of health records [13,37,38]	Evaluating and applying	Persistent avoidance of delabeled drugs; re-labeling; continued use of broad-spectrum alternatives [5,6]	Structured written summary after delabeling; updating of health records; follow-up communication [37,38]

DHR, drug hypersensitivity reaction; HL, health literacy.
